# A case of tacrolimus-induced supraventricular arrhythmia after kidney transplantation

**DOI:** 10.1590/1516-3180.2013.1313472

**Published:** 2013-06-01

**Authors:** Bo-Ra Kim, Ho-Sik Shin, Yeon-Soon Jung, Hark Rim

**Affiliations:** I MD. Fellowship, Department of Internal Medicine, Kosin University College of Medicine, Busan, Korea.; II MD. Assistant Professor, Kosin University College of Medicine, Gospel Hospital, Busan, Korea.; III MD, PhD. Associate Professor, Department of Internal Medicine, Kosin University College of Medicine, Busan, Korea.; IV MD, PhD. Professor, Department of Internal Medicine, Kosin University College of Medicine, Busan, Korea.

**Keywords:** Tacrolimus, Arrhythmias, cardiac, Kidney transplantation, Tachycardia, supraventricular, Atrial premature complexes, Tacrolimo, Arritimias cardíacas, Transplante de rim, Taquicardia supraventricular, Complexos atriais prematuros

## Abstract

**CONTEXT:**

Tacrolimus is a potent immunosuppressive drug often administered to transplant recipient patients and exhibits a variety of adverse cardiovascular effects.

**CASE REPORT:**

We report a case of a 53-year-old Asian female who developed various arrhythmic phenomena including atrial premature complexes and supraventricular tachycardia after administration of tacrolimus.

**CONCLUSION:**

Tacrolimus-associated arrhythmia after kidney transplantation may be life-threatening, and so patients undergoing this procedure should be carefully monitored.

## RESUMO

**CONTEXTO:**

Tacrolimus (tacrolimo) é uma potente droga imunossupressora frequentemente administrada a pacientes receptores de transplante e exibe uma variedade de efeitos cardiovasculares adversos.

**RELATO DE CASO:**

Relatamos um caso de uma mulher asiática de 53 anos de idade que desenvolveu vários fenômenos de arritmias, incluindo complexos atriais prematuros e taquicardia supraventricular após a administração de tacrolimus.

**CONCLUSÃO:**

Tacrolimus associado a arritmia após transplante de rim pode ser fatal, por isso, pacientes submetidos a esse procedimento devem ser cuidadosamente monitorados.

## INTRODUCTION

Tacrolimus, a potent immunosuppressive drug often administered to transplant recipients, exhibits a variety of adverse cardiovascular effects. The common symptoms are chest pain and hypertension, and abnormal electrocardiographic findings have also been reported.^1^^,^^2^ Supraventricular tachycardia (SVT) has never before been reported as an adverse effect of tacrolimus in adults. Here, we present a case of tacrolimus-induced supraventricular arrhythmia after renal transplantation.

## CASE REPORT

The patient was a 53-year-old woman with immunoglobulin A nephropathy resulting in end-stage renal failure. After ten years of hemodialysis, she underwent cadaveric kidney transplantation. The patient had no past history and no family history of cardiac dysrhythmia, syncope or sudden death. An electrocardiogram one day prior to surgery showed normal sinus rhythm, and an echocardiogram showed mild cardiomegaly with normal systolic function.

**Figure 1.**
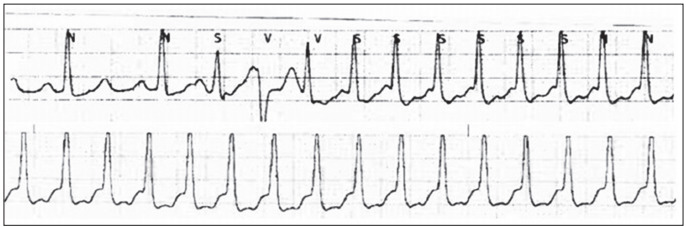
Electrocardiogram (EKG) on the patient: ventricular premature beats were detected (upper trace). Soon after the ventricular ectopic beat, supraventricular tachycardia developed (lower trace).

After the operation, methylprednisolone, mycophenolate mofetil and tacrolimus were administered. The tacrolimus dose was gradually increased from 3 mg twice a day, and sporadic atrial premature beats were detected on the second postoperative day (POD 2). The tacrolimus blood concentration had increased to 10.6 ng/ml. The tacrolimus dose was decreased, and the blood concentration then decreased to close to 8 ng/ml. On POD 3, the incidence increased significantly, accompanied by appearance of SVT and sporadic ventricular premature beats (Figure 1). The tacrolimus blood concentration was measured and had again increased to 17.9 ng/ml. The SVT was controlled with amiodarone and beta blocker. The reduced drug dose was continued, and the blood concentration was maintained close to 8 ng/ml; arrhythmia did not recur at this level. In both episodes, electrolytes (sodium, potassium and calcium) were within normal ranges (Table 1). QT and QTc were 400/500 ms on POD 2 and 374/467 ms on POD 3. In our case, the QTc data and tacrolimus concentration were not correlated.

**Table 1.** Serum chemistry, QT interval and tacrolimus concentration

Day	QT	QTc	Potassium (mEq/l)	Calcium (mg/dl)	Tacrolimus concentration (ng/ml)
Pre-op	442	493	4.5	9.0	
POD 1	404	526	4.4	8.1	
POD 2	376	487	4.7	8.4	10.6
POD 3	400	500	4.5	8.6	8.8
POD 4	396	469	4.4	9.1	17.6

Pre-op = preoperative; POD = postoperative day.

## DISCUSSION

Tacrolimus is one of the most frequently used immunosuppressive agents, and arrhythmia is a little-recognized adverse effect. Tacrolimus is a macrolide compound that is considered to prolong QT intervals and result in *torsades de pointes*.^3^ A systematic survey of indexed articles using the MeSH (Medical Subject Headings) terms “tacrolimus” and “arrhythmia” in databases revealed that only five articles have been published on this topic to this date. All of these papers were found in the Medline, Embase, Lilacs and Cochrane Library databases (Table 2).^1^^,^^2^^,^^3^^,^^4^^,^^5^

**Table 2.** Results from our reviews of medical databases using descriptors for the main clinical findings observed in our patient: October 26, 2012

Data base	Search strategy	Results
PubMed	“Tacrolimus” AND “arrhythmia”	4 case reports^1-4^ 1 review of the literature^5^
Embase	“Tacrolimus” AND “arrhythmia”	4 case reports^1-4^ 1 review of the literature^5^
Lilacs	“Tacrolimus” AND “arrhythmia”	4 case reports^1-4^ 1 review of the literature^5^
Cochrane Library	“Tacrolimus” AND “arrhythmia”	4 case reports^1-4^ 1 review of the literature^5^

Johnson first reported prolonged QT intervals and torsades de pointes after tacrolimus administration in a ten-year old patient who underwent liver transplantation.^1^ A second report involved a 35-year-old woman with a long history of renal failure due to systemic lupus erythematosus.^2^ In both cases, tacrolimus was infused intravenously to more rapidly increase the drug concentration. It was thus suggested that use of intravenous tacrolimus might be temporally related to these tachyarrhythmias. The third reported case involved a one-year-old girl who underwent liver transplantation in whom, despite a comparatively low concentration of tacrolimus, various arrhythmic phenomena including atrial premature beats, SVT and ventricular tachycardia (VT) were observed. Tacrolimus was orally administered in that case; however, the patient had hepatic dysfunction, which appeared to affect the metabolism of tacrolimus and prolong the half-life of the drug.^4^ Recently, ventricular arrhythmia occurring in kidney transplant recipients was reported, with the prevalence of associated factors. In the multiple logistic regression analysis, male gender and coronary artery calcification score were independently associated with the presence of ventricular arrhythmia.^5^

In our case, the patient was an adult female, and the coronary artery calcification score was not evaluated. However, the electrocardiogram showed normal sinus rhythm, and the echocardiogram showed mild cardiomegaly with normal systolic function. In addition, tacrolimus was administered orally and there was no indication of hepatic dysfunction or other causes that would reduce the half-life of the drug. The maximum tacrolimus blood concentration was 17.9 ng/ml, i.e. within the therapeutic range (5-20 ng/ml). Tacrolimus is a commonly used immunosuppressant, and patients in whom it is administered should be carefully monitored for cardiac dysrhythmia.

## CONCLUSION

After kidney transplantation, a 53-year-old woman developed tacrolimus-induced supraventricular arrhythmia. Tacrolimus-associated arrhythmia may be life-threatening and patients who take the drug, intravenously or even orally, should be carefully monitored.
